# Efficacy and Safety of Glucagon-Like Peptide-1 Receptor Agonists for Obesity Management in Adults With and Without Type 2 Diabetes: A Systematic Review

**DOI:** 10.1155/jobe/3897161

**Published:** 2025-10-19

**Authors:** Jena Velji-Ibrahim, Dhruvil Radadiya, Kalpit Devani, Harsh Patel, Piyush Nathani, Cesare Hassan, Nicola Pugliese, Christopher Thompson, Prateek Sharma

**Affiliations:** ^1^Gastroenterology and Liver Center, Prisma Health-Upstate, University of South Carolina School of Medicine, Greenville, South Carolina, USA; ^2^Gastroenterology, Cedars-Sinai Medical Center, Los Angeles, California, USA; ^3^Department of Gastroenterology, Hepatology and Motility, University of Kansas Medical Center, Kansas City, Kansas, USA; ^4^Department of Gastroenterology and Hepatology, Humanitas Research Hospital, Milan, Italy; ^5^Department of Advanced Endoscopy, Brigham and Women's Hospital, Boston, Massachusetts, USA; ^6^Department of Gastroenterology, Kansas City VA Medical Center, Kansas City, Missouri, USA

**Keywords:** diabetes mellitus, glucagon-like peptide-1 receptor agonist, obesity, tirzepatide, weight loss

## Abstract

**Objective:**

This systematic review aimed to assess the efficacy and safety of GLP-1 RAs in adults with obesity or overweight, by comparing different GLP-1 RAs, identifying the most effective agents, and evaluating adverse effects.

**Methods:**

We systematically searched Embase, MEDLINE, and Cochrane for phase 3 and 4 randomized controlled trials (RCTs) with a minimum duration of 40 weeks. Included studies compared GLP-1 RAs to placebo or to each other in adults with obesity (BMI ≥ 30 kg/m^2^) or overweight (BMI ≥ 27 kg/m^2^), with or without type 2 diabetes (T2DM). We excluded crossover trials, open-label studies, early-phase trials, and studies focusing on specific subpopulations.

**Results:**

A total of 22 RCTs involving 41,757 participants were included. Among adults with T2DM, the greatest weight reductions were observed with tirzepatide 15 mg (−9.5 kg at 40 weeks; 72% lost ≥ 5% of baseline weight) and semaglutide 2.4 mg (−9.6% body weight at 68 weeks; 69% lost ≥ 5%). In participants without T2DM, semaglutide 2.4 mg (−14.9% body weight at 68 weeks) and tirzepatide 15 mg (−20.9% at 72 weeks) produced the most substantial effects, while semaglutide 50 mg was also effective in nondiabetic patients. Liraglutide 3 mg showed modest efficacy. Across trials, GLP-1 RAs were consistently associated with a higher frequency of gastrointestinal adverse events compared to placebo, including nausea (14%–28% vs. 5%–10%), vomiting (6%–12% vs. 2%–4%), and diarrhea (8%–20% vs. 4%–7%). The risk of pancreatitis and serious adverse events remained comparable to placebo.

**Conclusions:**

GLP-1 RAs, especially semaglutide and tirzepatide, are effective for weight management. Liraglutide may remain a viable, cost-effective alternative.

**Summary**

• What is already known on this topic: GLP-1 receptor agonists are increasingly used for weight loss, but their effectiveness and safety require further evaluation.
• What this study adds: In this systematic review of 22 randomized controlled trials with 41,757 individuals, semaglutide 2.4 mg and tirzepatide 5 mg, 10 mg, and 15 mg were consistently effective for weight loss in individuals with and without diabetes. Semaglutide 50 mg was also effective in adults without type 2 diabetes, while liraglutide 3 mg showed modest efficacy, making it a potential cost-effective alternative. GLP-1 RAs were associated with gastrointestinal side effects, but the risk of pancreatitis and serious adverse events was comparable to placebo.
• How this study might affect research, practice, or policy: GLP-1 receptor agonists offer an effective obesity treatment, particularly for individuals who struggle with lifestyle-based weight loss. Understanding their relative efficacy and side effect profiles helps guide personalized obesity management strategies. Future studies should explore long-term metabolic effects, safety profiles, and potential benefits beyond weight reduction.

## 1. Introduction

The treatment of obesity extends beyond weight reduction; it requires addressing the underlying pathophysiologic mechanisms exacerbated by obesity, which contribute to complications, comorbidities, and mortality. These complications may include type 2 diabetes (T2DM), dyslipidemia, arterial hypertension, metabolic dysfunction–associated steatotic liver disease (MASLD), and obstructive sleep apnea (OSA) [1]. Effective obesity management is a critical component in preventing T2DM and reducing the risk of cardiovascular disease or advanced chronic liver disease [2, 3]. Beyond weight loss, emerging data demonstrate metabolic benefits of obesity therapies, including reduction in hepatic steatosis, improvements in endothelial function, and favorable effects on heart failure [4, 5].

Efforts to reverse the obesity epidemic encompass interventions including lifestyle changes, pharmacotherapy, endoscopic interventions, and surgery. Comprehensive lifestyle modifications, including diet and exercise, are the cornerstone of all weight management interventions [6, 7]. However, the available evidence shows that the resulting weight loss of 1–6 kg is challenging to maintain [8–10]. For such individuals, antiobesity medications may be used as an adjunctive pharmacotherapy.

Glucagon-like peptide-1 receptor agonists (GLP-1 RAs), recognized for their antidiabetic properties, have been investigated as antiobesity drugs. They increase insulin secretion, reduce appetite, delay gastric emptying, and modulate dopamine reward pathways to decrease cravings and food intake [9, 11, 12]. These medications bind to GLP-1 receptors in the central nervous system, pancreas, and intestines to regulate hunger and satiety [9, 12]. The US Food and Drug Administration (FDA) has approved daily liraglutide 3 mg, weekly semaglutide 2.4 mg, and recently, weekly tirzepatide for obesity management [13–15]. Tirzepatide, a dual glucose-dependent insulinotropic polypeptide (GIP) and GLP-1 RA, enhances insulin secretion and sensitivity while acting on additional nutrient-stimulated hormone pathways [11, 15–17]. The availability of high-dose GLP-1 RAs in both oral and injectable formulations offers promising individualized treatment options.

While clinical trials of varying sizes have been conducted to investigate the weight loss efficacy of these drugs, individual studies have varied in design and population and have reported inconsistent weight loss effects [10, 11, 17–32]. Few head-to-head studies have compared the efficacy and safety of GLP-1 RAs. There is also substantial variability regarding weight loss in patients with diabetes compared to patients without diabetes. This systematic review aimed to provide an overview of the weight loss efficacy and adverse event profile of GLP-1 RAs in two populations: adults with overweight/obesity with and without T2DM.

## 2. Methods

### 2.1. Overview

We initially aimed to conduct a network meta-analysis to compare the efficacy and safety of GLP-1 RAs for obesity management, integrating direct and indirect evidence from multiple randomized controlled trials (RCTs). However, substantial heterogeneity across trials, including variations in study design, participant characteristics, and outcome measures, made this approach unfeasible. The lack of direct head-to-head RCT comparisons between GLP-1 RAs further hindered a robust network meta-analysis. As a result, we opted for a narrative systematic review to present findings from each study, organizing key trial features and population characteristics that may influence drug effectiveness and interpretation.

### 2.2. Data Sources and Searches

Embase, MEDLINE, and Cochrane were systematically searched from inception to April 1, 2025, for trials assessing GLP-1 RA efficacy and safety in weight management in adults with overweight/obesity, both with and without T2DM. Preferred Reporting Items for Systematic Reviews and Meta-Analyses (PRISMA) guidelines were followed [33]. The protocol for this study was registered on PROSPERO (CRD42023466527). Search terms included in our literature search are shown in eTable 1. We restricted the search to clinical trials published in the English language and involving human subjects. Risk of bias for individual studies was assessed using the Cochrane Risk of Bias 2 tool (Supporting Information, eFigure 2). To minimize the potential for publication bias, we conducted a comprehensive search of multiple databases and reviewed reference lists of included studies and relevant review articles.

### 2.3. Study Selection

Our inclusion criteria for clinical trials were as follows: (1) phase 3 or 4 RCTs comparing GLP-1 RAs to each other or placebo; (2) minimum treatment duration of 40 weeks to assess efficacy and safety; (3) participants aged 18 or older; (4) BMI ≥ 30 kg/m^2^ or ≥ 27 kg/m^2^ with obesity-related comorbidities (e.g., hypertension, dyslipidemia, OSA, and cardiovascular disease); and (5) primary or secondary outcome of change in body weight. We excluded open-label, crossover, phase 1 or 2 trials, and studies focused on specific populations like polycystic ovary syndrome.

### 2.4. Data Extraction and Synthesis

In this systematic review, absolute weight change and percentage total body weight loss (%TBWL) were the primary efficacy outcomes. Efficacy benchmarks were considered met if the mean weight loss difference between the treatment and placebo groups was at least 5%, or if the proportion of patients losing ≥ 5% of baseline body weight in the active group was at least 35% and approximately double that of the placebo group [34]. Secondary outcomes focused on safety, including gastrointestinal side effects (nausea, vomiting, diarrhea, and constipation), pancreatitis, and serious adverse events (as defined by the Medical Dictionary for Regulatory Activities). Side effect data were extracted and presented as risk differences (RDs) with 95% confidence intervals (CIs). RD was chosen to directly compare the proportion of adverse events between treatment and control groups, allowing for a clearer interpretation of the absolute risk increase or decrease associated with GLP-1 RA therapy. Although baseline characteristics were extracted, subgroup analyses based on these variables were not conducted.

### 2.5. Quality Assessment

Two reviewers (Jena Velji-Ibrahim and Dhruvil Radadiya) independently extracted data from the RCTs using a standardized form, recording study details, sample size, efficacy, safety outcomes, GLP-1 RA type, dose, and follow-up period. Baseline characteristics (age, sex, BMI, waist circumference, and A1c) were also noted. Conflicts were resolved by consultation with the third author (Kalpit Devani). To minimize bias and ensure accurate reporting, we consulted Qureshi et al. [35]. Two reviewers assessed the risk of bias using the Cochrane Risk of Bias Tool Version 2 [36], with any conflicts resolved by Kalpit Devani.

### 2.6. Role of Funding Source

The authors did not receive any funding or financial support for any part of the study. Therefore, our study and its design are free from any influence by the pharmaceutical companies that manufacture the drugs compared in this analysis.

### 2.7. Ethics Statement

This systematic review was confirmed by the Human Investigation Committee (IRB) of Prisma Health to be exempt from IRB approval because it utilized only publicly available, de-identified data from previously published RCTs and did not involve direct interaction with human subjects or access to private, identifiable information.

## 3. Results

The study selection process is shown in eFigure 1 and eTable 1. A total of 22 RCTs were included, with durations ranging from 40 to 160 weeks and sample sizes from 282 to 17,604 participants. All trials were double-blinded. 18 RCTs evaluated the efficacy of a GLP-1 RA compared with placebo: daily liraglutide 3 mg (5 RCTs) [21, 22, 27, 28, 37], daily liraglutide 1.8 mg (3 RCTs) [21, 22, 29], weekly efpeglenatide 2 mg (1 RCT) [38], weekly efpeglenatide 4 mg (1 RCT) [38], weekly efpeglenatide 6 mg (1 RCT) [38], weekly albiglutide 30 mg (1 RCT) [39], weekly albiglutide 50 mg (1 RCT) [39], daily semaglutide 25 mg (1 RCT) [18], daily semaglutide 50 mg (2 RCTs) [18, 32], weekly semaglutide 2.4 mg (5 RCTs) [10, 24, 26, 40, 41], weekly tirzepatide 5 mg (2 RCTs) [17, 20], and weekly tirzepatide 10 and 15 mg (3 RCTs) [17, 20, 30]. Other comparisons included multidose weekly dulaglutide [42], weekly semaglutide 2.4 mg versus daily liraglutide 3 mg [19], weekly semaglutide 2.4 mg versus weekly semaglutide 1 mg [24], multidose weekly tirzepatide with weekly semaglutide 1 mg [25], and multidose weekly tirzepatide with weekly dulaglutide [31].

All but one of the included studies were Phase 3 RCTs, with a Phase 4 RCT by Pi-Sunyer and colleagues [27]. Most studies used subcutaneous injections, except for two that utilized oral semaglutide [16, 32]. A total of 41,757 participants (with or without T2DM) with obesity (BMI ≥ 30 kg/m^2^ or ≥ 27 kg/m^2^ with obesity-related comorbidities) were included. Participant ages ranged from 45 to 58 years, with 38.7% male participants. Baseline characteristics, including body weight, BMI, and HbA1c, are summarized in Table 1.

### 3.1. Weight Loss in Participants With Diabetes

**Figure 1:**
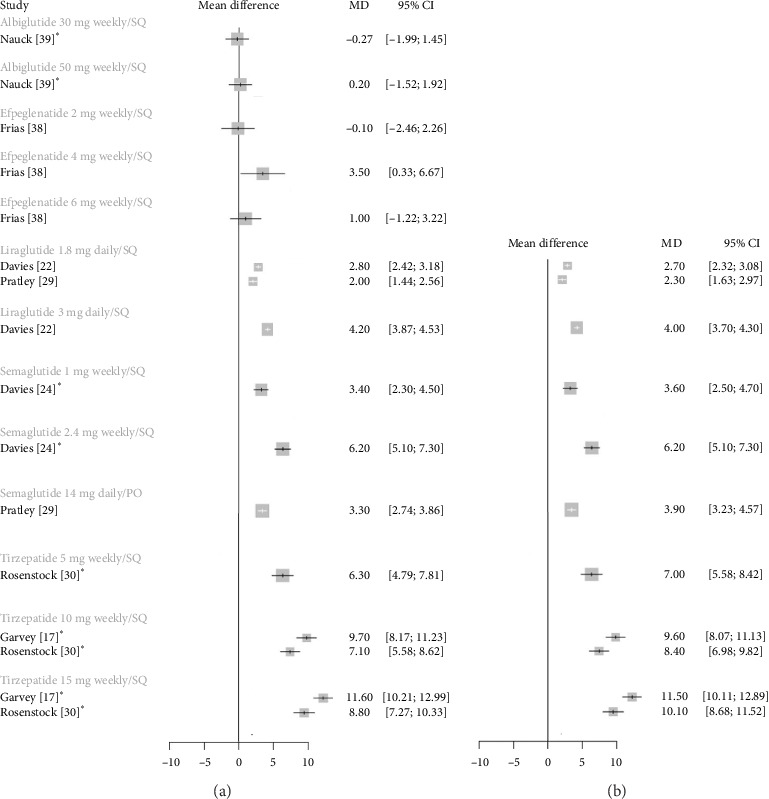
Forest plots for mean difference of weight loss from baseline with GLP-1 RAs versus placebo in a diabetic population. (a) Absolute weight change (kg). (b) Total body weight loss (%TBWL). An asterisk denotes the trials that included structured lifestyle interventions. Each study arm is shown separately for comparison. Error bars represent 95% confidence intervals. Abbreviations: %TBWL = percentage total body weight loss, GLP-1 RAs = glucagon-like peptide-1 receptor agonists, kg = kilograms, and MD = mean difference.

Figure 1 illustrates the absolute weight change and %TBWL with each GLP-1 RA compared to placebo in participants with diabetes. Significant weight loss was observed with all GLP-1 RAs except efpeglenatide and albiglutide. Tirzepatide 15 mg was associated with the most weight loss, followed by tirzepatide 10 mg. Weight loss with tirzepatide 5 mg and semaglutide 2.4 mg was similar. Efficacy benchmarks were met if the difference in mean weight loss between the treatment and placebo groups was at least 5%, or if at least 35% of patients lost ≥ 5% of baseline body weight, approximately double that of the placebo group [34].

#### 3.1.1. Albiglutide

A 52-week double-blind trial found no significant weight loss difference between albiglutide (30 mg and 50 mg) and placebo [39]. Neither efficacy benchmark was met. Participants had a lower BMI, with 33 kg/m^2^ in the placebo group and 34 kg/m^2^ in the albiglutide groups, compared to other studies. Males comprised 57% in the placebo and albiglutide 30 mg group and 51% in the albiglutide 50 mg group. Participants received diet and exercise counseling at each study visit.

#### 3.1.2. Efpeglenatide

Efpeglenatide was studied in a 56-week trial that did not include lifestyle modifications. Here, the baseline BMI of participants was 34 kg/m^2^. Efpeglenatide was associated with significant weight loss compared to placebo, but the effect slightly decreased from weeks 30–56 [38]. Neither benchmark was met in this study.

#### 3.1.3. Dulaglutide

One study demonstrated that higher doses of dulaglutide (3.0 mg and 4.5 mg) resulted in substantially greater weight loss than the 1.5 mg dose. However, both benchmarks were not met. Lifestyle modifications were not part of the trial protocol. This trial had a near-equal percentage of male and female participants. Participants had an average BMI of 34 kg/m^2^. They were permitted to continue metformin use.

#### 3.1.4. Liraglutide

The satiety and clinical adiposity-liraglutide evidence (SCALE) Diabetes trial, a 56-week phase 3a study, compared various doses of liraglutide to placebo. The absolute weight changes were 6.4 kg with liraglutide 3 mg, 5.0 kg with liraglutide 1.8 mg, and 2.2 kg with placebo [22]. The mean weight loss difference (< 5%) did not meet the efficacy benchmark, but > 35% of subjects in the liraglutide group lost ≥ 5% of baseline body weight. Lifestyle interventions included dietary counseling and 150 min of weekly physical activity. This study only had 21% and 22% males in the GLP-1 RA group and placebo group, respectively. Insulin was used as rescue medication.

#### 3.1.5. Semaglutide

The semaglutide treatment effect in people with obesity (STEP) 2 trial, a double-blind, double-dummy, phase 3 superiority study, showed that both efficacy benchmarks were met with semaglutide 2.4 mg [24]. A significant proportion (68.6%) of patients lost ≥ 5% of baseline body weight. Lifestyle interventions included dietary counseling and 150 min of weekly physical activity. Insulin use as rescue medication was not allowed.

Only one study compared high doses of oral semaglutide in diabetics, comparing semaglutide 25 mg and 50 mg to the 14 mg dose [18]. Over 35% of participants lost ≥ 5% of weight with high doses. Lifestyle modifications were not included, and participants could continue other oral T2DM medications.

#### 3.1.6. Tirzepatide

The study of tirzepatide in participants with T2DM (SURPASS) 1 trial, the shortest at 40 weeks, compared tirzepatide to placebo and reported weight losses of 7.0 kg, 7.8 kg, and 9.5 kg with tirzepatide 5 mg, 10 mg, and 15 mg, respectively. This was the only study where both weight loss benchmarks were met, although participants had a lower baseline BMI of 32 kg/m^2^ compared to 35–37 kg/m^2^ in other studies [30]. There were a similar number of male and female participants. In a 72-week double-blind, randomized, placebo-controlled study, both benchmarks were met with tirzepatide 10 mg and 15 mg [17]. This was the only study that allowed participants to continue using insulin and oral T2DM medications, improving the generalizability to patients who start a GLP-1 RA while already on insulin. Lifestyle interventions were similar to the SCALE and STEP 2 trials, including dietary counseling and 150 min of physical activity per week.

#### 3.1.7. Comparison of Different Medications

Three studies directly compared different GLP-1 RAs. A double-blind, double-dummy study compared oral semaglutide 14 mg daily to liraglutide 1.8 mg daily and placebo. Although the weight loss difference between GLP-1 RAs and placebo was < 5%, 44.7% of participants taking oral semaglutide lost ≥ 5% of baseline body weight [29]. Adherence was a concern due to different administration routes. Participants were permitted to take metformin or an SGLT2 inhibitor. An open-label, 40-week phase 3 trial comparing tirzepatide 5 mg, 10 mg, and 15 mg to oral semaglutide 1 mg showed that > 35% of participants lost ≥ 5% of baseline body weight in all groups. However, only tirzepatide 15 mg met both benchmarks, as considerable weight loss was achieved with semaglutide 1 mg. Blinding was challenging due to device differences and dose-escalation schemes [25]. Participants were allowed to take metformin. The SURPASS J-mono trial, a double-blind, multicenter, randomized phase 3 study in Japanese patients, compared tirzepatide to dulaglutide, showing that both benchmarks were met with tirzepatide, though the homogeneous population may limit generalizability [31]. This study included participants with the lowest BMI of all studies, being in the overweight, not obese range.

### 3.2. Weight Loss in Participants Without Diabetes

**Figure 2:**
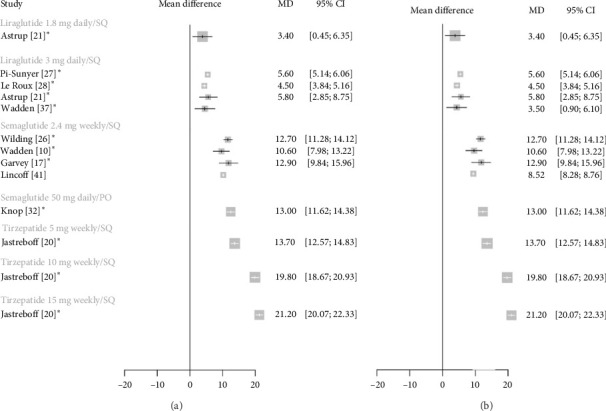
Forest plots for mean difference of weight loss from baseline with GLP-1 RAs versus placebo in a nondiabetic population. (a) Absolute weight change (kg). (b) Total body weight loss (%TBWL). An asterisk denotes the trials that included structured lifestyle interventions. Each study arm is shown separately for comparison. Error bars represent 95% confidence intervals. Abbreviations: %TBWL = percentage total body weight loss, GLP-1 RAs = glucagon-like peptide-1 receptor agonists, kg = kilograms, and MD = mean difference.

Figure 2 illustrates absolute weight change and %TBWL with each GLP-1 RA compared to placebo in a population without diabetes. All GLP-1 RAs were associated with significant weight loss compared to placebo. Tirzepatide 15 mg was associated with the most weight loss, followed closely by tirzepatide 10 mg. Similar weight loss was achieved with tirzepatide 5 mg, semaglutide 2.4 mg, and oral semaglutide 50 mg.

#### 3.2.1. Liraglutide

Four trials compared liraglutide 3 mg to placebo, one of which included liraglutide 1.8 mg [21]. Two were phase 3a [27, 28] and two were phase 3b trials [21, 37]. In all studies, > 35% of participants lost ≥ 5% of baseline body weight; however, only two studies met both efficacy benchmarks with a 5% mean difference in weight loss between groups [21, 27]. Astrup and colleagues [21] and Le Roux and colleagues [28] provided long-term efficacy data (160 weeks). Both studies included lifestyle modifications, but Wadden and colleagues [37] integrated intensive behavioral therapy, which may limit generalizability. This study showed considerable placebo group weight loss, reducing the difference between groups. All three studies had predominantly female populations, with Wadden and colleagues including 16% and 17% males in the liraglutide and placebo groups, respectively [21, 28, 37]. Additionally, Le Roux and colleagues only included participants with prediabetes [28].

#### 3.2.2. Semaglutide

One multicenter, double-blind study compared semaglutide 2.4 mg to placebo without lifestyle modifications in participants without T2DM. This trial, the largest in terms of participants, lasted 104 weeks and had mostly male participants (72%), who were older (average age 62 years), and had the lowest average participant BMI of 33 kg/m^2^ [41].

Three trials (STEP 1, 3, and 5) compared semaglutide 2.4 mg to placebo and included lifestyle modifications [10, 26, 40]. STEP 1 had the largest sample size [26], while STEP 3, with intensive behavioral therapy, demonstrated the smallest weight loss difference [10]. All three studies had a mostly female population. While STEP 1 and 3 were 68 weeks, STEP 5 was 104 weeks. These trials were pivotal for semaglutide market approval, as efficacy benchmarks were met with all trials.

One study compared oral semaglutide 50 mg to placebo over 68 weeks, aligning with STEP 1 and 3 durations [32]. This study included lifestyle modifications and had a mostly female population.

#### 3.2.3. Tirzepatide

A single 72-week study comparing tirzepatide to placebo also met both benchmarks [20]. Participants in this study had a similar baseline BMI and percentage of male participants to the other trials in nondiabetics.

#### 3.2.4. Comparison of Different Medications

The randomized, open-label, phase 3b STEP 8 study compared semaglutide 2.4 mg to liraglutide 3 mg directly in individuals without T2DM [19]. It found that semaglutide resulted in significantly greater weight loss compared to liraglutide in adults with overweight or obesity. The weight loss and adverse effect profiles of semaglutide and liraglutide were similar to those reported by Wilding and colleagues [26] and Pi-Sunyer and colleagues [27].

### 3.3. Side Effects

**Figure 3:**
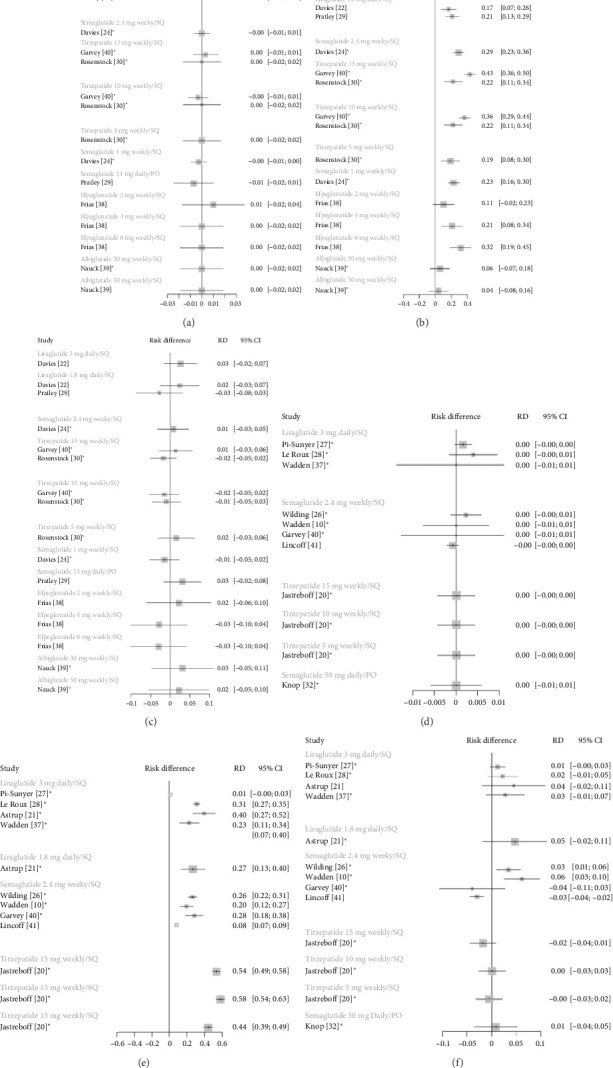
Adverse effects with GLP-1 RAs versus placebo in adults with and without type 2 diabetes. Adverse effects are presented as risk difference. (a)–(c) illustrate pancreatitis (a), gastrointestinal side effects (b), and serious adverse events (c) in a diabetic population. (d)–(f) exhibit pancreatitis (d), gastrointestinal side effects (e), and serious adverse events (f) in a nondiabetic population. Each study arm is shown separately for direct comparison. Abbreviations: GLP-1 RA = glucagon-like peptide-1 receptor agonist; RD = risk difference.

Figure 3 illustrates the RD of various side effects of GLP-1 RAs compared to placebo. In both the T2DM and non-T2DM populations, there was no greater risk of pancreatitis with GLP-1 RAs compared to placebo. In both diabetic and nondiabetic populations, all GLP-1 RAs except for albiglutide and efpeglenatide 2 mg were more likely than placebo to be associated with gastrointestinal adverse effects such as nausea, vomiting, diarrhea, and constipation. Tirzepatide showed comparable RDs across all doses. The RD of serious adverse events was comparable between GLP-1 RAs and placebo.

### 3.4. Heterogeneity of Trials

The inability to pool data in our analysis is due to the heterogeneity among the included studies. Differences in study design, population characteristics, GLP-1 RA doses, treatment duration, and the presence of lifestyle interventions contributed to this variation. For instance, some trials focused on individuals with T2DM, while others included nondiabetic participants, leading to differences in baseline characteristics like BMI and comorbidities. The studies also used various GLP-1 RAs at different doses, including daily and weekly formulations, complicating direct comparisons. Additionally, the inclusion of lifestyle interventions such as diet and exercise varied, influencing weight loss outcomes. The use of rescue medications, like insulin or oral T2DM drugs, further affected generalizability, preventing data pooling for a unified analysis.

## 4. Discussion

In this systematic review encompassing 22 RCTs with 41,757 adults, we aimed to provide an overview of the efficacy and safety of GLP-1 RAs for weight management in adults with and without T2DM. Our findings suggest that while most GLP-1 RAs offer substantial weight loss benefits, both efficacy benchmarks were met if the mean weight loss versus placebo was ≥ 5%, or if ≥ 35% of participants in the treatment group lost ≥ 5% of baseline body weight. Using these criteria, semaglutide 2.4 mg and tirzepatide 5 mg, 10 mg, and 15 mg met efficacy benchmarks in both populations, while semaglutide 50 mg met benchmarks in adults without T2DM. We found a comparable safety profile between GLP-1 RAs, although a higher incidence of gastrointestinal side effects was observed with liraglutide 3 mg.

Liraglutide 3 mg, while showing modest weight loss, has not consistently met efficacy benchmarks compared to newer GLP-1 RAs such as semaglutide and tirzepatide [24, 26, 40]. However, it has demonstrated benefits in reducing major cardiovascular events, reducing the risk of new-onset persistent macroalbuminuria, and slowing the decline in estimated glomerular filtration rate in patients with T2DM [43]. With its patent expiration and the availability of a generic form, liraglutide may become a more cost-accessible option despite its lower efficacy. This shift might increase its use despite its relatively lower efficacy, highlighting the ongoing need to balance cost, accessibility, and clinical outcomes in obesity management.

Beyond weight loss, GLP-1 RAs have beneficial systemic effects. They reduce the risk of major adverse cardiovascular events and preserve renal function, including reductions in kidney failure and slowing the decline in estimated glomerular filtration rate [4]. Emerging evidence also suggests that GLP-1 RAs reduce hepatic steatosis and liver inflammation, which is particularly relevant given the high prevalence of MASLD and MASH in individuals with obesity [5, 44].

Previous studies, and this systematic review, confirm that individuals with T2DM face greater challenges in losing weight compared to those without diabetes. Although semaglutide 2.4 mg and all doses of tirzepatide met weight loss benchmarks in both T2DM and non-T2DM populations, weight reduction was generally less pronounced in adults with T2DM. This may reflect lower baseline body weight, concomitant use of medications such as sulfonylureas and insulin that promote weight gain [2], reduced glycosuria, altered gut microbiome, and genetic predisposition to weight gain [45, 46]. Additionally, individuals with T2DM may be older and may have struggled with obesity for longer than those without T2DM.

Emerging therapies offer potential improvements in efficacy and broader treatment options, particularly for patients with diabetes, where obesity's pathophysiology is more complex. For example, orforglipron, a once-daily, oral, nonpeptide GLP-1 RA, may provide a competitive alternative to oral semaglutide as it does not require fasting [47]. Notably, retatrutide has demonstrated superior weight loss compared to tirzepatide in early phase trials [48]. By activating GLP-1, GIP, and glucagon receptors, it exerts effects across multiple metabolic pathways, regulating appetite, energy expenditure, and glucose homeostasis, which may provide enhanced efficacy, particularly in patients with obesity and T2DM. Retatrutide represents a promising advancement in pharmacologic therapy for obesity, offering potential for greater weight reduction than currently available GLP-1 RAs, although long-term safety and real-world effectiveness remain under investigation. Overall, new agents may improve efficacy while addressing a wider range of therapeutic needs and patient preferences, offering more tailored options for managing obesity and its comorbidities.

Limitations to the intertrial comparisons include the variability in lifestyle interventions, with some studies including intensive behavioral therapy while others did not incorporate formal lifestyle modifications [10, 37]. Real-world studies of adherence to these lifestyle interventions are needed, as these may not be representative of adherence in the general population. Given the variability in follow-up duration, shorter follow-up periods in certain trials may underestimate long-term efficacy and safety. Although formal assessment of publication bias was limited by the small number of studies for each GLP-1 RA and dose, we minimized potential bias by conducting a comprehensive search of multiple databases and reviewing reference lists of included studies and relevant reviews.

Demographics should also be considered when interpreting results [49]. For example, STEP 2, SURMOUNT-2, and SURPASS-1 included 51%, 51%, and 48% female participants, respectively. However, STEP 3, STEP 4, and SURMOUNT-1 included 81%, 79%, and 68% female participants, respectively. This discrepancy is important as females have been reported to respond more favorably to GLP-1 RAs, with some studies suggesting greater weight loss in females compared to males [50, 51]. Additionally, while STEP 1 and STEP 2 were designed to have a greater proportion of Asians than other trials [52], they still had a predominantly Caucasian population. One study included a 100% Asian population [31]. Future research should focus on underrepresented populations and gender-based differences to improve generalizability.

Considering safety, similar gastrointestinal side effects across GLP-1 RAs suggest these adverse events are inherent to the class and linked to their mechanisms of action. Previous RCTs and meta-analyses reported higher gastrointestinal side effects with subcutaneous semaglutide compared to liraglutide [11, 19]. Our systematic review, which assessed safety alongside efficacy, found similar gastrointestinal side effects across GLP-1 RAs, with liraglutide 3 mg showing the highest incidence. Notably, GLP-1 RAs had a similar incidence of pancreatitis and serious adverse events compared to placebo, consistent with previous meta-analyses [11, 16].

In conclusion, this systematic review highlights the efficacy and safety of GLP-1 RAs for weight management, and our findings support the use of GLP-1 RAs, particularly semaglutide 2.4 mg and all doses of tirzepatide, as highly effective strategies for weight management in adults with and without diabetes. Semaglutide 50 mg was also effective in the nondiabetic population. As liraglutide is off-patent, its reduced cost could increase its accessibility. Future research should prioritize head-to-head comparisons, long-term cardiovascular and hepatic outcomes, and personalized approaches considering gender and ethnicity.

## Figures and Tables

**Table 1:** Overview of studies included in this systematic review and participant baseline characteristics.

Study	GLP-1 RA/comparator	Frequency/route	*n*	Duration (weeks)	Lifestyle interventions	T2DM population	Age (years)	Male (%)	Baseline weight (kg)	BMI (kg/m^2^)	WC (cm)	HbA1c (%)	BP (mm Hg)	TC (mg/dl)	HDL (mg/dl)	LDL (mg/dl)	TG (mg/dl)	eGFR	Additional information
Albiglutide																			
Nauck [39]	Albiglutide 30 mg	Weekly/SQ	102	52	Dietary + exercise counseling	Yes	54	57	96	34	—	8.0	—	—	—	—	—	—	
	Albiglutide 50 mg	Weekly/SQ	102	52	Dietary + exercise counseling	Yes	52	51	97	34	—	8.2	—	—	—	—	—	—	
	Placebo	Weekly/SQ	105	52	Dietary + exercise counseling	Yes	53	57	96	33	—	8.0	—	—	—	—	—	—	
Dulaglutide																			
Frias [42]	Dulaglutide 1.5 mg	Weekly/SQ	612	52	None	Yes	58	49	96	34	—	8.6	132/79	—	—	—	—	93	Metformin permitted
	Dulaglutide 3 mg	Weekly/SQ	616	52	None	Yes	57	53	96	34	—	8.6	131/78	—	—	—	—	93	Metformin permitted
	Dulaglutide 4.5 mg	Weekly/SQ	614	52	None	Yes	57	52	96	34	—	8.6	132/79	—	—	—	—	94	Metformin permitted
Efpeglenatide																			
Frias [38]	Efpeglenatide 2 mg	Weekly/SQ	100	56	None	Yes	59	55	98	34	—	8.1	—	—	—	—	—	100	
	Efpeglenatide 4 mg	Weekly/SQ	101	56	None	Yes	56	52	95	34	—	8.1	—	—	—	—	—	100	
	Efpeglenatide 6 mg	Weekly/SQ	103	56	None	Yes	60	59	96	34	—	8.1	—	—	—	—	—	100	
	Placebo	Weekly/SQ	102	56	None	Yes	60	50	98	35	—	8.0	—	—	—	—	—	95	
Liraglutide																			
Astrup [21]	Liraglutide 1.8 mg	Daily/SQ	90	52	500 kcal/day deficit diet + increased physical activity	No	46	24	99	35	108	5.6	130/82	—	—	—	—	—	
	Liraglutide 3 mg	Daily/SQ	93	52	500 kcal/day deficit diet + increased physical activity	No	46	25	99	35	109	5.6	131/82	—	—	—	—	—	
	Placebo	Daily/SQ	98	52	500 kcal/day deficit diet + increased physical activity	No	46	25	98	35	108	5.6	128/81	—	—	—	—	—	
Davies [22]	Liraglutide 1.8 mg	Daily/SQ	211	56	None	Yes	55	51	106	37	118	8.0	131/80	178	45	92	170	—	Metformin, thiazolidinediones, &/or sulfonylureas permitted
	Liraglutide 3 mg	Daily/SQ	423	56	None	Yes	55	52	106	37	118	7.9	129/79	171	45	86	162	—	Metformin, thiazolidinediones, &/or sulfonylureas permitted
	Placebo	Daily/SQ	212	56	None	Yes	55	45	107	37	117	7.9	129/79	169	45	85	158	—	Metformin, thiazolidinediones, &/or sulfonylureas permitted
Le Roux [28]	Liraglutide 3 mg	Daily/SQ	1505	160	Dietary + exercise counseling	No	48	24	108	39	117	5.8	125/79	193	50	112	133	—	Excluded participants without prediabetes
	Placebo	Daily/SQ	749	160	Dietary + exercise counseling	No	47	23	108	39	117	5.7	125/80	197	50	116	133	—	Excluded participants without prediabetes
Pi-Sunyer [27]	Liraglutide 3 mg	Daily/SQ	2487	56	Dietary + exercise counseling	No	45	21	106	38	115	5.6	123/79	194	51	112	126	—	
	Placebo	Daily/SQ	1244	56	Dietary + exercise counseling	No	45	22	106	38	115	5.6	123/79	194	51	112	129	—	
Pratley [29]	Liraglutide 1.8 mg	Daily/SQ	284	52	None	Yes	56	52	96	33	109	8.0	—	—	—	—	—	96	Metformin &/or SGLT2 inhibitor permitted
	Placebo	Daily/SQ	142	52	None	Yes	57	52	93	33	108	7.9	—	—	—	—	—	95	Metformin &/or SGLT2 inhibitor permitted
	Semaglutide 14 mg	Daily/PO	285	52	None	Yes	56	52	93	33	108	8.0	—	—	—	—	—	96	Metformin &/or SGLT2 inhibitor permitted
Wadden [37]	Liraglutide 3 mg	Daily/SQ	142	56	Calorie-based diet; 100 mins of physical activity/week, increased to 250 mins after 24 weeks	No	45	16	109	39	116	5.5	125/80	190	112	50	58	—	
	Placebo	Daily/SQ	140	56	Calorie-based diet; 100 mins of physical activity/week, increased to 250 mins after 24 weeks	No	49	17	107	39	115	5.5	127/81	197	120	54	54	—	
Semaglutide																			
Aroda [18]	Semaglutide 25 mg	Daily/PO	535	68	None	Yes	59	57	97	34	113	9.0	133/80	—	—	—	—	—	Metformin, sulfonylurea, &/or SGLT2 inhibitor permitted
	Semaglutide 50 mg	Daily/PO	535	68	None	Yes	58	57	96	34	112	8.9	132/80	—	—	—	—	—	Metformin, sulfonylurea, &/or SGLT2 inhibitor permitted
Davies [24]	Semaglutide 1.0 mg	Weekly/SQ	403	68	500 kcal/day deficit diet + 150 mins of physical activity/week	Yes	56	50	99	35	114	8.1	130/80	—	—	—	—	93	Participants on metformin &/or SGLT2 inhibitor; insulin permitted for persistent hyperglycemia
	Semaglutide 2.4 mg	Weekly/SQ	404	68	500 kcal/day deficit diet + 150 mins of physical activity/week	Yes	55	45	99	36	115	8.1	130/80	—	—	—	—	94	Participants on metformin &/or SGLT2 inhibitor; insulin permitted for persistent hyperglycemia
	Placebo	Weekly/SQ	403	68	500 kcal/day deficit diet + 150 mins of physical activity/week	Yes	55	53	100	36	116	8.1	130/80	—	—	—	—	92	Participants on metformin &/or SGLT2 inhibitor; insulin permitted for persistent hyperglycemia
Knop [32]	Semaglutide 50 mg	Daily/PO	334	68	500 kcal/day deficit diet + 150 mins of physical activity/week	No	49	26	105	37	113	5.6	129/82	193	50	116	124	96	
	Placebo	Daily/PO	333	68	500 kcal/day deficit diet + 150 mins of physical activity/week	No	50	29	106	38	115	5.6	130/83	190	50	108	124	93	
Garvey [40]	Semaglutide 2.4 mg	Weekly/SQ	152	104	Dietary + exercise counseling	No	47	19	106	39	116	5.7	126/80	190	46	112	50	96	
	Placebo	Weekly/SQ	152	104	Dietary + exercise counseling	No	47	26	107	39	116	5.7	125/80	186	46	112	46	93	
Lincoff [41]	Semaglutide 2.4 mg	Weekly/SQ	8803	104	None	No	62	72	97	33	111	5.8	131/80	153	44	78	134	82	
	Placebo	Weekly/SQ	8801	104	None	No	62	72	97	33	111	5.8	131/80	153	44	78	135	82	
Rubino [19]	Semaglutide 2.4 mg	Weekly/SQ	126	68	500 kcal/day deficit diet + 150 mins of physical activity/week	No	48	24	103	37	112	5.5	125/81	185	52	106	110	96	No masking due to dose differences
	Liraglutide 3 mg	Daily/SQ	127	68	500 kcal/day deficit diet + 150 mins of physical activity/week	No	49	30	104	37	114	5.5	126/81	189	54	108	113	95	No masking due to dose differences
Wadden [10]	Semaglutide 2.4 mg	Weekly/SQ	407	68	Low-calorie diet 8 weeks, then hypocaloric diet + 100 min/week physical activity, increased to 200 min/week after 16 weeks	No	46	23	107	38	114	5.7	124/80	185	52	108	108	97	
	Placebo	Weekly/SQ	204	68	Low-calorie diet 8 weeks, then hypocaloric diet + 100 min/week physical activity, increased to 200 min/week after 16 weeks	No	46	12	104	38	114	5.8	124/81	189	51	112	111	97	
Wilding [26]	Semaglutide 2.4 mg	Weekly/SQ	1306	68	500 kcal/day deficit diet + 150 mins of physical activity/week	No	46	27	105	38	115	5.7	126/80	190	49	110	126	96	
	Placebo	Weekly/SQ	655	68	500 kcal/day deficit diet + 150 mins of physical activity/week	No	47	24	105	38	115	5.7	127/80	192	49	113	128	96	
Tirzepatide																			
Frias [42]	Tirzepatide 5 mg	Weekly/SQ	470	40	None	Yes	56	44	93	34	108	8.3	131/79	172	43	88	166	97	Participants on metformin; blinding limited by device and dose-escalation differences
	Tirzepatide 10 mg	Weekly/SQ	469	40	None	Yes	57	51	95	34	111	8.3	131/80	171	43	88	167	96	Participants on metformin; blinding limited by device and dose-escalation differences
	Tirzepatide 15 mg	Weekly/SQ	470	40	None	Yes	56	45	94	35	110	8.3	130/79	169	43	86	164	96	Participants on metformin; blinding limited by device and dose-escalation differences
	Semaglutide 1 mg	Weekly/SQ	469	40	None	Yes	57	48	94	34	109	8.3	130/79	171	43	88	165	96	Participants on metformin; blinding limited by device and dose-escalation differences
Garvey [17]	Tirzepatide 10 mg	Weekly/SQ	312	72	500 kcal/day deficit diet + 150 mins of physical activity/week	Yes	54	49	101	36	114	8.0	131/80	178	46	97	186	96	Oral T2DM medication or insulin permitted
	Tirzepatide 15 mg	Weekly/SQ	311	72	500 kcal/day deficit diet + 150 mins of physical activity/week	Yes	54	49	100	36	115	8.1	130/80	174	43	93	177	96	Oral T2DM medication or insulin permitted
	Placebo	Weekly/SQ	315	72	500 kcal/day deficit diet + 150 mins of physical activity/week	Yes	55	50	102	37	116	7.9	131/80	178	43	101	186	94	Oral T2DM medication or insulin permitted
Inagaki [31]	Tirzepatide 5 mg	Weekly/SQ	159	52	None	Yes	57	71	79	29	97	8.2	130/82	195	51	108	151	78	Multicenter in Japan; adults > 20 years old; T2DM treatment washed out over 8 weeks
	Tirzepatide 10 mg	Weekly/SQ	158	52	None	Yes	56	75	79	28	96	8.2	130/83	191	50	104	154	80	Multicenter in Japan; adults > 20 years old; T2DM treatment washed out over 8 weeks
	Tirzepatide 15 mg	Weekly/SQ	160	52	None	Yes	56	83	79	28	97	8.2	132/84	187	49	103	146	80	Multicenter in Japan; adults > 20 years old; T2DM treatment washed out over 8 weeks
	Dulaglutide 0.75 mg	Weekly/SQ	159	52	None	Yes	58	74	77	28	95	8.2	131/82	191	51	108	138	79	Multicenter in Japan; adults > 20 years old; T2DM treatment washed out over 8 weeks
Jastreboff [20]	Tirzepatide 5 mg	Weekly/SQ	630	72	500 kcal/day deficit diet + 150 mins of physical activity/week	No	46	32	103	37	113	5.6	124/80	187	48	109	129	98	
	Tirzepatide 10 mg	Weekly/SQ	636	72	500 kcal/day deficit diet + 150 mins of physical activity/week	No	45	33	106	38	115	5.6	124/80	191	48	112	127	98	
	Tirzepatide 15 mg	Weekly/SQ	630	72	500 kcal/day deficit diet + 150 mins of physical activity/week	No	45	33	106	38	114	5.6	123/79	187	48	110	128	98	
	Placebo	Weekly/SQ	643	72	500 kcal/day deficit diet + 150 mins of physical activity/week	No	44	32	105	38	114	5.6	123/80	186	47	109	131	98	
Rosenstock [30]	Tirzepatide 5 mg	Weekly/SQ	121	40	Dietary + exercise counseling	Yes	54	46	87	32	104	8.0	128/80	181	43	101	154	95	
	Tirzepatide 10 mg	Weekly/SQ	121	40	Dietary + exercise counseling	Yes	56	60	86	32	103	7.9	128/79	180	43	101	149	92	
	Tirzepatide 15 mg	Weekly/SQ	121	40	Dietary + exercise counseling	Yes	53	52	85	32	103	7.9	127/79	184	43	105	149	96	
	Placebo	Weekly/SQ	115	40	Dietary + exercise counseling	Yes	54	49	85	32	102	8.1	128/80	178	43	97	152	93	

Abbreviations: BMI = body mass index, BP = blood pressure, eGFR = estimated glomerular filtration rate, HbA1c = glycated hemoglobin, HDL = high-density lipoprotein cholesterol, LDL = low-density lipoprotein cholesterol, *n* = number of participants, PO = oral administration, SQ = subcutaneous administration, T2DM = type 2 diabetes mellitus, TC = total cholesterol, TG = triglycerides, and WC = waist circumference.

## Data Availability

Data and study materials will be available to other researchers by emailing the corresponding author.

## Supporting Information

Supporting Information 1 Supporting File 1: this file contains the completed PRISMA (Preferred Reporting Items for Systematic Reviews and Meta-Analyses) checklist, documenting adherence to standardized reporting guidelines for systematic reviews and meta-analyses.

Supporting Information 2 Supporting File 2: this supporting file includes additional materials supporting the main manuscript. eTable 1 outlines the detailed search strategy used for database queries. eFigure 1 presents a flowchart summarizing the study selection process. eFigure 2 displays the risk of bias assessment for the included randomized controlled trials.
