# Correction to “Efficacy and Safety of Glucagon‐Like Peptide‐1 Receptor Agonists for Obesity Management in Adults With and Without Type 2 Diabetes: A Systematic Review”

**DOI:** 10.1155/jobe/9821679

**Published:** 2026-02-03

J. V.‐Ibrahim, D. Radadiya, K. Devani, et al., “Efficacy and Safety of Glucagon‐Like Peptide‐1 Receptor Agonists for Obesity Management in Adults With and Without Type 2 Diabetes: A Systematic Review,” *Journal of Obesity* (2025) 2025: 3897161, https://doi.org/10.1155/jobe/3897161.

In the article titled, “Efficacy and Safety of Glucagon‐Like Peptide‐1 Receptor Agonists for Obesity Management in Adults With and Without Type 2 Diabetes: A Systematic Review,” author Cesare Hassan was affiliated to Department of Gastroenterology and Hepatology, Humanitas Research Hospital, Milan, Italy which is incorrect. The correct affiliations for this author are as follows:

^1^Endoscopy Unit, IRCCS Humanitas Research Hospital, Milano, Italy

^2^Department of Biomedical Sciences, Humanitas University, Milano, Italy.

The corrected list of affiliations is shown in the author information below.

Jena Velji‐Ibrahim^1^, Dhruvil Radadiya^2^, Kalpit Devani^1^, Harsh Patel^3^, Piyush Nathani^3^, Cesare Hassan^4,5^, Nicola Pugliese^6^, Christopher Thompson^7^, Prateek Sharma^3,8^

^1^Gastroenterology and Liver Center, Prisma Health‐Upstate, University of South Carolina School of Medicine, South Carolina, Greenville, USA

^2^Gastroenterology, Cedars‐Sinai Medical Center, California, Los Angeles, USA

^3^Department of Gastroenterology, Hepatology and Motility, University of Kansas Medical Center, Kansas, Kansas City, USA

^4^Endoscopy Unit, IRCCS Humanitas Research Hospital, Milano, Italy

^5^Department of Biomedical Sciences, Humanitas University, Milano, Italy

^6^Department of Gastroenterology and Hepatology, Humanitas Research Hospital, Milan, Italy

^7^Department of Advanced Endoscopy, Brigham and Women’s Hospital, Massachusetts, Boston, USA

^8^Department of Gastroenterology, Kansas City VA Medical Center, Missouri, Kansas City, USA

We apologize for this error.

