# Supplementary material for: Efficacy and Safety of Glucagon-Like Peptide-1 Receptor Agonists for Obesity Management in Adults With and Without Type 2 Diabetes: A Systematic Review
Source: J Obes. 2025 Oct 19;2025:3897161. doi: 10.1155/jobe/3897161 (PMC12591819; doi:10.1155/jobe/3897161)

**Supplementary Online Content**

**eTable 1**. Search strategy for database searches

**eFigure 1**. Flowchart summarizing study selection.

**eFigure 2**. Bias assessment of included randomized control trials

**eTable 1**. Search strategy for database searches.

| **No.** | **Databases** | **Search Terms** | **Results**  **Total = 3,524** |
| --- | --- | --- | --- |
| **1** | **MEDLINE** | (obesity OR obesity management OR anti-obesity agents) OR (body weight OR body weight changes OR overweight OR “overweight” OR obese) OR (Body Mass Index OR BMI) AND (Glucagon-like peptide-1 OR GLP-1 agonist OR GLP-1 OR tirzepatide OR semaglutide OR dulaglutide OR liraglutide OR exenatide OR lixisenatide OR efpeglenatide OR albiglutide) AND “Clinical Trial” [Publication Type] OR "clinical trials as topic"[MeSH Terms] OR "clinical trial"[All Fields]) | **1,376** |
| **2** | **Embase** | (obesity OR obesity management OR anti-obesity agents) OR (overweight OR “overweight” OR obese) OR (Body Mass Index) AND (Glucagon-like peptide-1 OR GLP-1 agonist OR GLP-1 OR tirzepatide OR semaglutide OR dulaglutide OR liraglutide OR exenatide OR lixisenatide OR efpeglenatide OR albiglutide) AND “Clinical Trial” | **1,214** |
| **3** | **Cochrane** | (obesity OR obesity management OR anti-obesity agents OR Body Mass Index) AND (Glucagon-like peptide-1 OR GLP-1 agonist OR GLP-1 OR tirzepatide OR semaglutide OR dulaglutide OR liraglutide OR exenatide OR lixisenatide OR efpeglenatide OR albiglutide) AND “Clinical Trial” | **934** |

**eFigure 1.** Flowchart summarizing study selection.

**
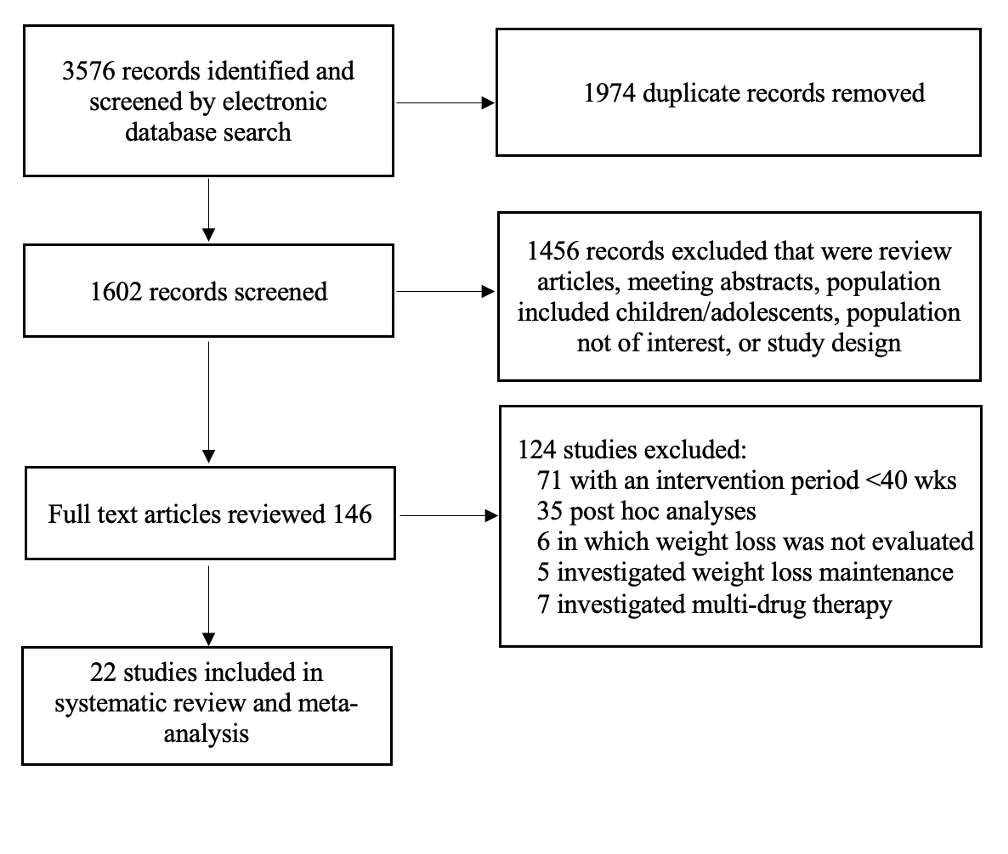
**

**eFigure 2**. Bias assessment of included randomized control trials. 2A includes the risk of bias assessment using Cochrane Risk of Bias 2 tool. 2B illustrates the overall risk of bias presented as a percentage of each risk of bias item across all included studies.

eFigure2A


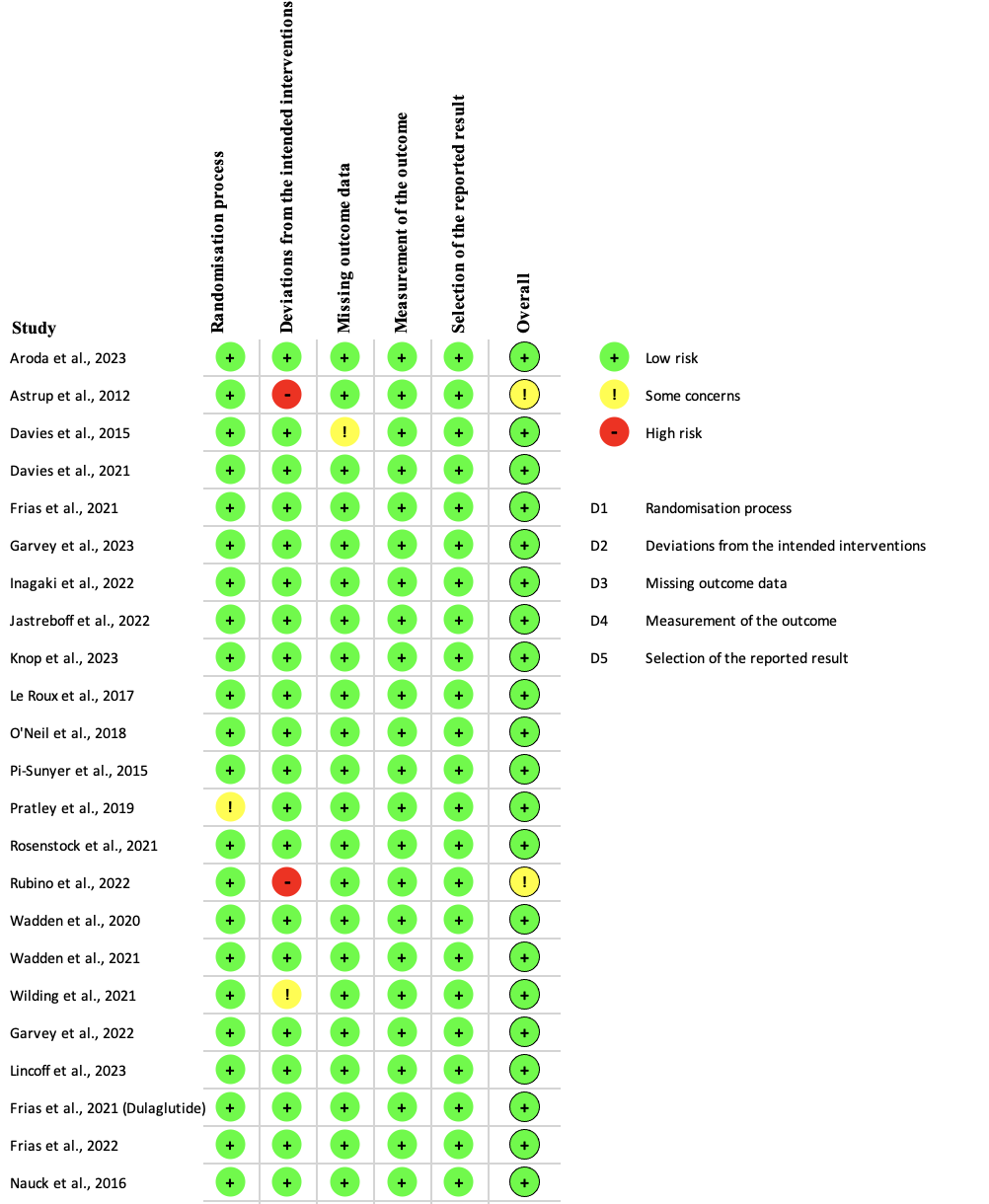


eFigure 2B


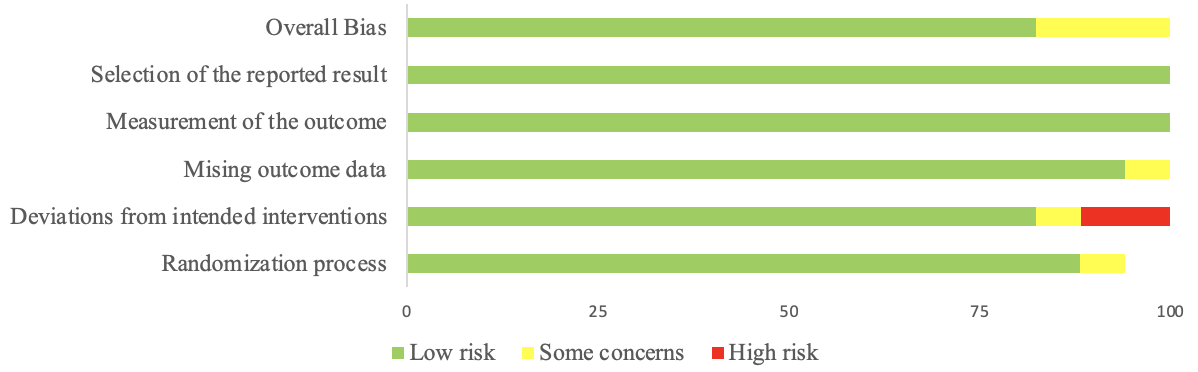

Supplement: Supporting Information 2 — Supporting File 2: this supporting file includes additional materials supporting the main manuscript. eTable 1 outlines the detailed search strategy used for database queries. eFigure 1 presents a flowchart summarizing the study selection process. eFigure 2 displays the risk of bias assessment for the included randomized controlled trials. [file 3897161.f2.docx]
